# Supplementary material for: Proteome-wide copy-number estimation from transcriptomics
Source: Mol Syst Biol. 2024 Sep 27;20(11):1230–56. doi: 10.1038/s44320-024-00064-3 (PMC11535397; doi:10.1038/s44320-024-00064-3)
Supplement: Supplementary file 1 — Appendix [file 44320_2024_64_MOESM1_ESM.pdf]

APPENDIX FOR

Proteome-wide copy-number estimation from transcriptomics

Andrew J. Sweatt[1], Cameron D. Griffiths[1], Sarah M. Groves[1], B. Bishal Paudel[1], Lixin Wang[1], David F. Kashatus[2], and Kevin A. Janes*[1,3]

Author affiliations: [1]Department of Biomedical Engineering, University of Virginia, Charlottesville, VA, USA, 22908; [2]Department of Microbiology, Immunology & Cancer Biology, University of Virginia, Charlottesville, VA, USA, 22908; [3]Department of Biochemistry & Molecular Genetics, University of Virginia, Charlottesville, VA, USA, 22908

Appendix Table of Contents

Appendix Text S1. Practical bounds on ∆CDF. 2

Appendix Supplementary References 3

Appendix Fig. S1. Shift-scale calibration of PaxDb and PTR to the meta-assembly. 4

Appendix Fig. S2. Relative predictions of Pinferna are closer to TMT proteomics than transcriptomics. 5

Appendix Fig. S3. Consensus re-clustering of 796 breast cancer cases from The Cancer Genome Atlas (TCGA) by protein inference. 6

Appendix Fig. S4. Poorer long-term prognosis for Luminal A breast cancers reclassified by Pinferna. 7

Appendix Fig. S5. Comparing transcriptomics- and Pinferna-based clusters of TCGA breast cancers. 8

Appendix Fig. S6. Inability to knock down NUP37 in luminal breast cancer lines. 9

Appendix Fig. S7. DNM1L dosage compensation limits overexpression in luminal breast cancer lines. 10

Appendix Text S1. Practical bounds on ΔCDF.

ΔCDF is a stringent metric for comparing model predictions against a rigorous null hypothesis—guessing a protein abundance based on prior measurements of that protein in other biological settings. Small absolute differences in ΔCDF are statistically separable because the metric considers the entire distribution of predictions rather than those of a single protein abundance (Methods). ΔCDF < 0 indicates predictions that are consistently worse than guessing and implies a proteome-wide bias in proportional copy numbers. Many of the competing methods yielded proteome predictions with ΔCDF < 0 (Fig. 3C,E).

In practice, we find that ~30% of the proteome is predicted equally well by any given method and the median null (|Scaled residual| < 0.5; Figs. 3A,B and EV3C,D and Appendix Text S1 Figure). Likewise, ~10% of the proteome is predicted poorly regardless (|Scaled residual| > 3; Figs. 3A,B and EV3C,D and Appendix Text S1 Figure). Prediction methods distinguish themselves by the 60% of genes in the middle. To calculate a practical maximum ΔCDF, we assumed no additional prediction error over that interval (|Scaled residual| = 0.5–3) and approximated the median null as an elliptical arc. Geometrically, this area is defined by $wh\left(1-\frac{\pi}{4}\right)$, where w is the width and h is the height of the interval. Substituting, we estimate that the practical maximum ΔCDF is (0.9 – 0.3)(3 – 0.5)(1 – π/4) = 0.32. Thus, the performance of Pinferna (ΔCDF = 0.15–0.2; Fig. 3C) is >50% of the way to the practical maximum.

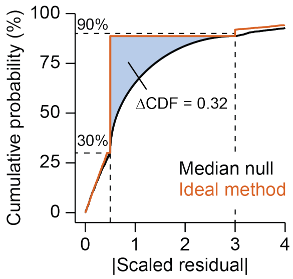

Idealized ΔCDF.

Appendix Supplementary References

Mertins P, Mani DR, Ruggles KV, Gillette MA, Clauser KR, Wang P, Wang X, Qiao JW, Cao S, Petralia F et al (2016) Proteogenomics connects somatic mutations to signalling in breast cancer. Nature 534:55-62

Zhang H, Liu T, Zhang Z, Payne SH, Zhang B, McDermott JE, Zhou JY, Petyuk VA, Chen L, Ray D et al (2016) Integrated Proteogenomic Characterization of Human High-Grade Serous Ovarian Cancer. Cell 166:755-765

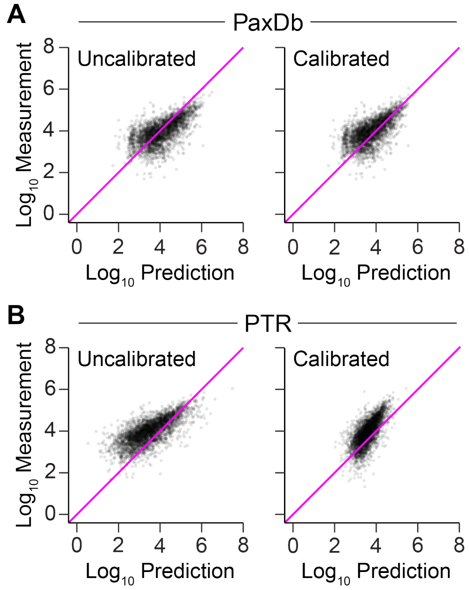

Appendix Fig. S1. Shift-scale calibration of PaxDb and PTR to the meta-assembly.

(A,B) Predictions for PaxDb **(A)** and PTR **(B)** in the meta-assembly were compared to SWATH-scaled proteomics before (left) and after (right) calibration to measurements as follows: $Y_{measurement} = aX^{b}_{uncalibrated}$, where *a* and *b* are shift and scale parameters estimated by maximizing ∆CDF of the calibration (Methods).

Data information: For **(A,B)**, *n* = 3213 **(A)** or 3771 **(B)** proteins predicted by each method for MDA-MB-468 cells as a representative example of the global fit .

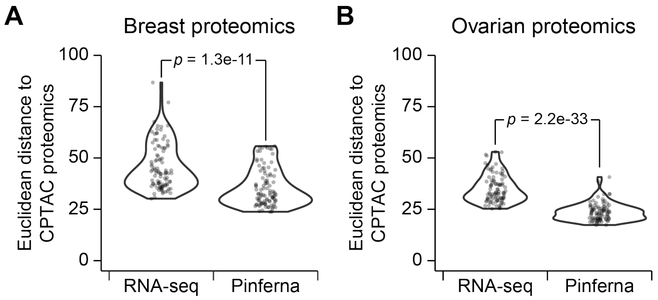

Appendix Fig. S2. Relative predictions of Pinferna are closer to TMT proteomics than transcriptomics.

(A,B) Euclidean distances between TMT data (log_2-centered by gene) and either RNA-seq data or Pinferna (log_2-centered by gene) were aggregated from the CPTAC breast cancer study of (Mertins et al, 2016) **(A)** or the CPTAC ovarian cancer study of (Zhang et al, 2016) **(B)**.

Data information: For **(A,B)**, n = 99 **(A)** or 93 **(B)** patient samples. Differences between groups were assessed by rank-sum test.

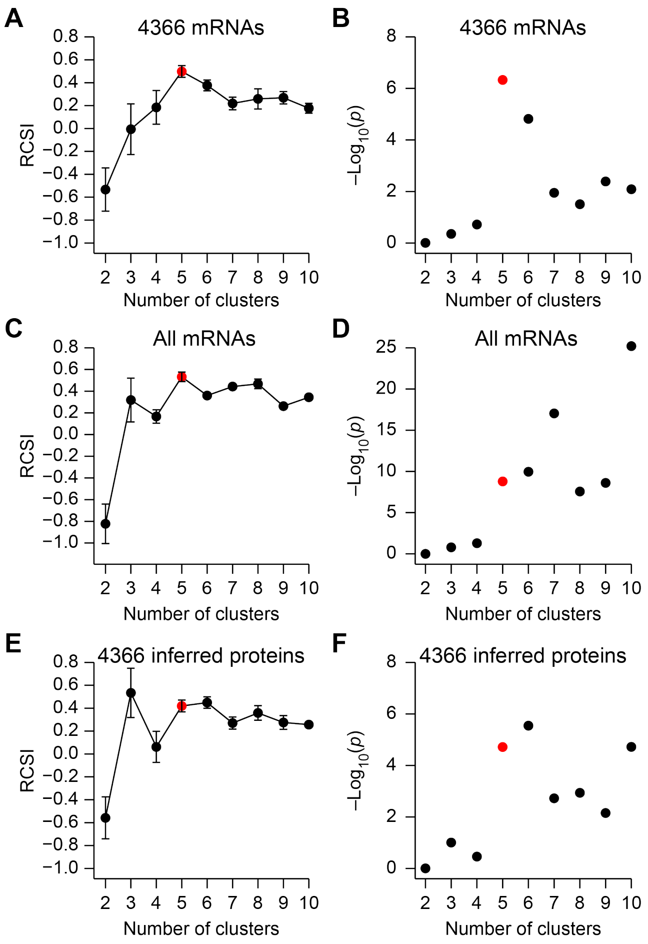

Appendix Fig. S3. Consensus re-clustering of 796 breast cancer cases from The Cancer Genome Atlas (TCGA) by protein inference.

(A–F) Relative cluster stability index (RCSI) **(A,C,E)** and significance **(B,D,F)** of cluster number for consensus clustering (Methods) applied to 4366 mRNAs used by Pinferna **(A,B)**, all mRNAs quantified by RNA-seq **(C,D)**, and protein inferences by Pinferna **(E,F)**. Stable maxima or near maxima at five clusters is indicated in red.

Data information: For **(A,C,E)**, RCSI is shown as the mean ± 95% confidence interval of n = 10 iterations of the consensus clustering algorithm.

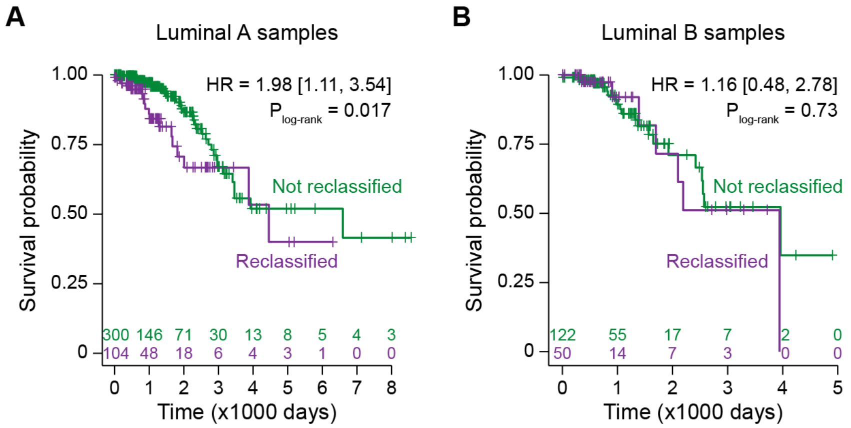

Appendix Fig. S4. Poorer long-term prognosis for Luminal A breast cancers reclassified by Pinferna.

TCGA breast tumors with **(A)** Luminal A PAM50 assignment and long-term outcome data or **(B)** Luminal B PAM50 assignment and long-term outcome data were stratified based on Pinferna-based protein-cluster reclassification status and compared by proportional hazards modeling (Methods).

Data information: For **(A,B)**, *n* = 404 **(A)** or 172 **(B)** cases. Differences were assessed by log-rank test, and the hazard ratio (HR) is shown with 95% confidence interval in brackets.

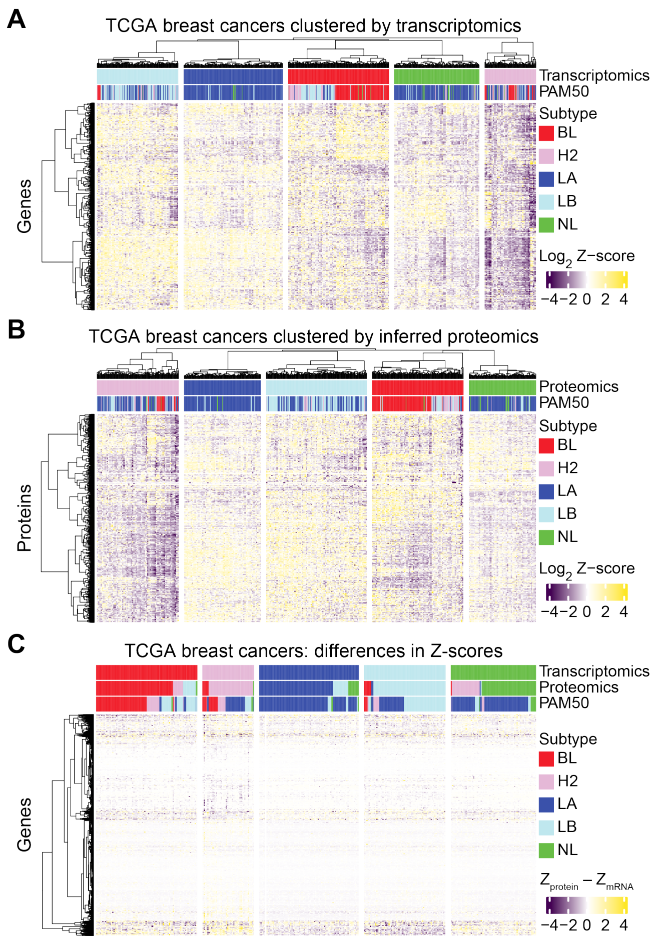

Appendix Fig. S5. Comparing transcriptomics- and Pinferna-based clusters of TCGA breast cancers.

(A,B) Two-way hierarchical clustering within transcriptomic **(A)** and inferred proteomic **(B)** clusters annotated as in Fig. 5A. **(C)** One-way hierarchical clustering of genes by differences in standardized Z-scores between inferred protein and mRNA ($Z_{protein} – Z_{mRNA}$).

Data information: For **(A–C)**, hierarchical clustering was performed by Euclidean distance and Ward's linkage. BL: Basal-like; H2: HER2+; LA: Luminal A; LB: Luminal B; NL: Normal-like.

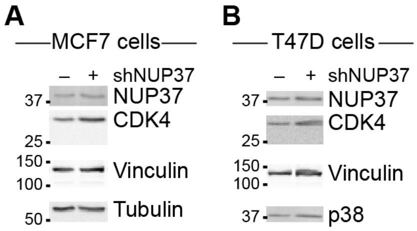

Appendix Fig. S6. Inability to knock down NUP37 in luminal breast cancer lines.

(A,B) MCF7 cells **(A)** or T47D cells **(B)** were stably transduced with doxycycline-inducible shNUP37 or shLuciferase control, induced with 1 µg/ml doxycycline for 48 hours, and immunoblotted for NUP37 or CDK4 with vinculin and tubulin or p38 as loading controls.

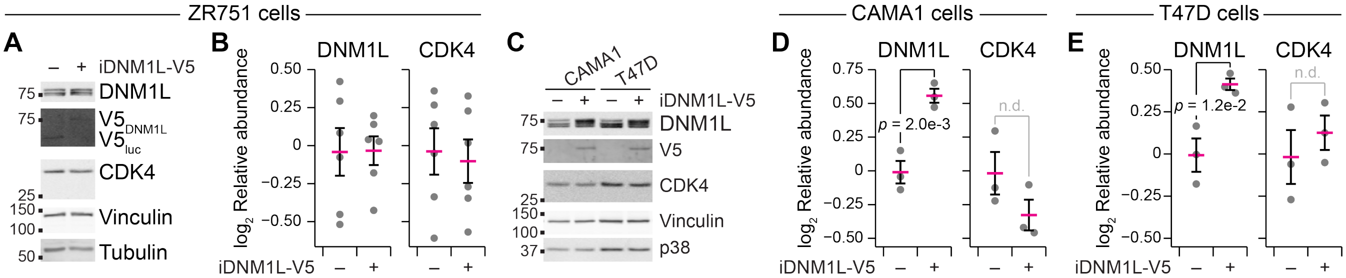

Appendix Fig. S7. DNM1L dosage compensation limits overexpression in luminal breast cancer lines.

(A,B) Inability to overexpress inducible DNM1L (iDNM1L). ZR751 cells were stably transduced with iDNM1L or luciferase control, induced with 1 µg/ml doxycycline for 24 hours, and immunoblotted for DNM1L, V5 epitope tag, and CDK4 with vinculin and tubulin as loading controls. For V5, image gamma = 4 to visualize weak-but-detectable bands. **(C–E)** Inability to overexpress DNM1L more than 1.5 fold. CAMA1 cells **(C,D)** or T47D cells **(C,E)** were stably transduced with iDNM1L or luciferase control, induced with 1 µg/ml doxycycline for 24 hours, and immunoblotted for DNM1L, V5 epitope tag, and CDK4 with vinculin and p38 as loading controls.

Data information: For **(B,D,E)**, immunoblot results are summarized as the mean total DNM1L (endogenous and induced) or CDK4 ± s.e.m. of n = 6 **(B)** or 3 **(D,E)** biological replicates. Differences were assessed by one-sided t test. n.d., no difference.
