# Supplementary material for: Proteome-wide copy-number estimation from transcriptomics
Source: Mol Syst Biol. 2024 Sep 27;20(11):1230–56. doi: 10.1038/s44320-024-00064-3 (PMC11535397; doi:10.1038/s44320-024-00064-3)
Supplement: Supplementary file 11 — Expanded View Figures [file 44320_2024_64_MOESM11_ESM.pdf]

Expanded View Figures

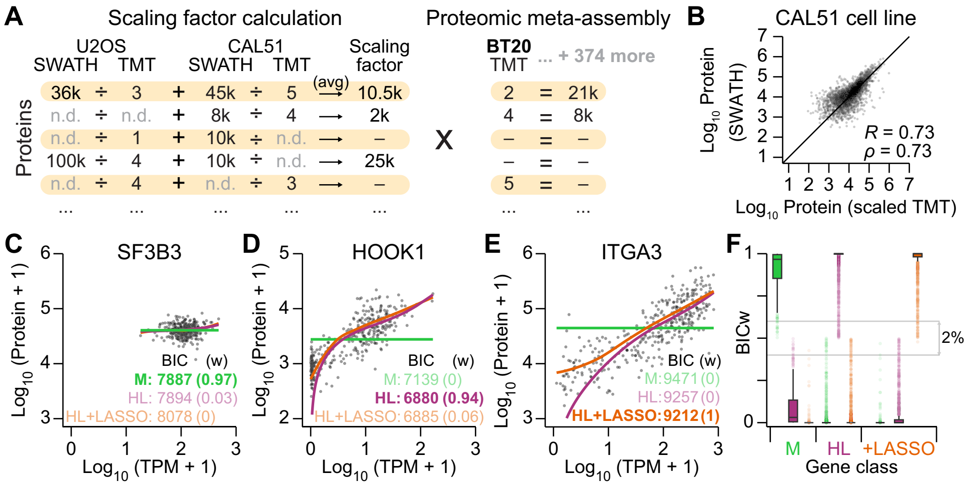

Figure EV1. Calibration for the proteomic meta-assembly and model selection examples.

(**A**) SWATH–TMT scaling factor estimation and calibration of the proteomic meta-assembly. Scaling factors were estimated for each protein by dividing the SWATH intensity by the TMT intensity in U2OS and CAL51 when both data types were available and averaging (avg) the two ratios when possible. The gene-specific scaling factors were used to convert the entire TMT dataset (bold) to proportional protein abundances. (**B**) The reciprocal cross-calibration to that shown in Fig. 1B. Step 1 of Fig. 1A was performed with U2OS data alone and the SWATH-scaled TMT proteomics of CAL51 cells compared with data obtained directly by SWATH. (**C–E**) Model selection of the representative genes shown in Fig. 1C–E. Proportional protein copies per cell were regressed against the mRNA abundance normalized as transcripts per million (TPM). Data are fit with M, HL, and HL + LASSO models. The BIC was used to discriminate the best model for each fit, and BIC weights (w) are shown in parentheses to indicate the relative best-model probability. The lowest BIC is indicated. (**F**) BIC weights (BICw) for each model fit to M, HL, or HL + LASSO (+ LASSO) genes. Range of weights for genes with model ambiguity (two models with a BICw ≥0.4) are boxed (gray) with the percentage indicated. Data information: For (**B**), Pearson's R and Spearman's ρ are shown. For (**C–E**), $n = 369$ cancer cell lines. For (**F**), box-and-whisker plots show the median BICw (horizontal line), interquartile range (IQR, box), and an additional 1.5 IQR extension from the box edge (whiskers) from $n = 4366$ genes.

© The Author(s)

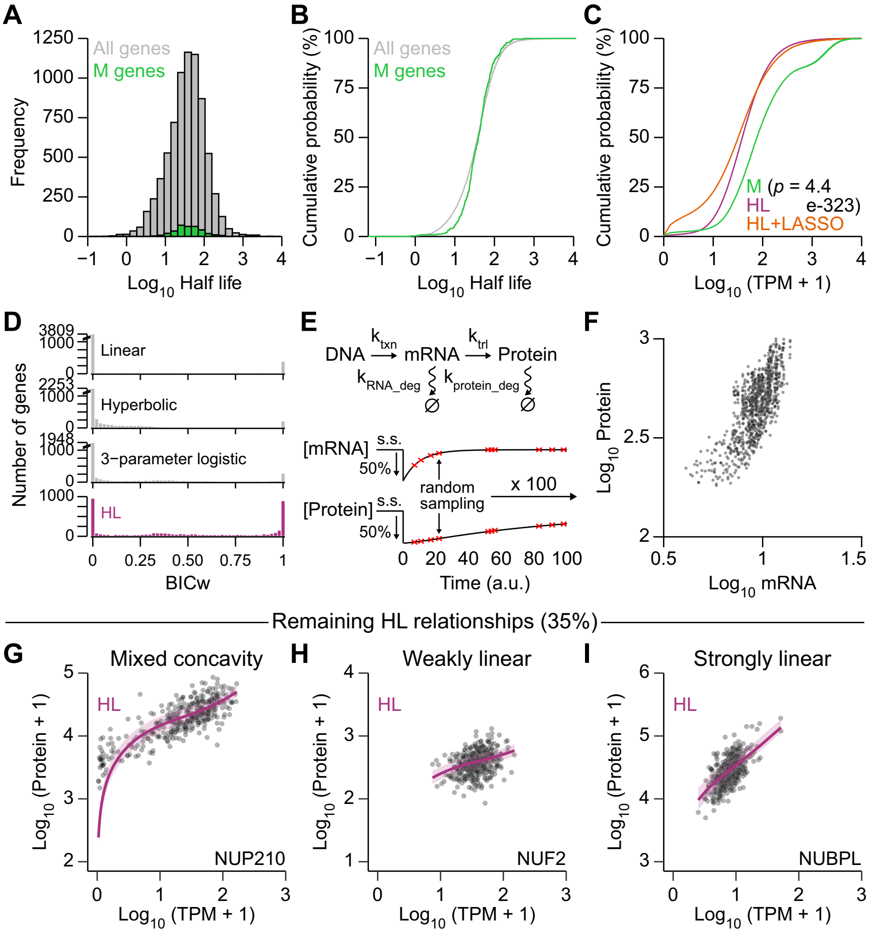

Figure EV2. Extended characterization of M and HL mRNA-to-protein relationship classes.

(**A**, **B**) M genes do not exhibit longer half-lives compared to other relationship classes. Half-lives for proteins were obtained from (Zecha et al, 2018) and plotted by frequency (**A**) or as an empirical cumulative distribution function (**B**) for all genes (gray) and M genes (green). (**C**) M genes are more abundant by mRNA compared to other relationship classes. mRNA abundance was normalized as TPM and placed on a log scale with a pseudocount of 1. Cumulative distribution functions are shown for M genes (green), HL genes (purple), and HL + LASSO genes (orange). (**D**) Distribution of BIC weights (BICw) for models encoding linear, hyperbolic, three-parameter logistic, and HL relationships shown in Fig. 2B. (**E**, **F**) Log-convex patterns arise when mRNA and protein abundances recover from transient perturbations to steady-state values. A simple transcription–translation model (**E**) was reduced by 50% and randomly sampled at 10 time points (red) during the return to steady state (s.s.). For the model, the following dimensionless rate parameters were used: $k_{txn} = 1$; $k_{trl} = 1$; $k_{RNA\_deg} = 0.1$; $k_{protein\_deg} = 0.01$. The model was simulated 100 times with lognormally distributed parameter noise (coefficient of variation = 10%) and the joint observation of mRNA and protein abundances ($n = 10$ time points x 100 simulations) is shown in (**F**). (**G–I**) Examples of other HL relationships besides those of Fig. 2C, D: mixed concavity (**G**), weakly linear (**H**), and strongly linear (**I**) relationships. Data information: For (**A**, **B**), $n = 7029$ genes (gray) and 334 genes (green). For (**C**), $n = 395$ genes (green), 2569 genes (purple), and 1402 genes (orange). Distributions were compared by K-S test with Šidák correction for multiple-hypothesis testing. For (**D**), $n = 4366$ genes.

 © The Author(s)

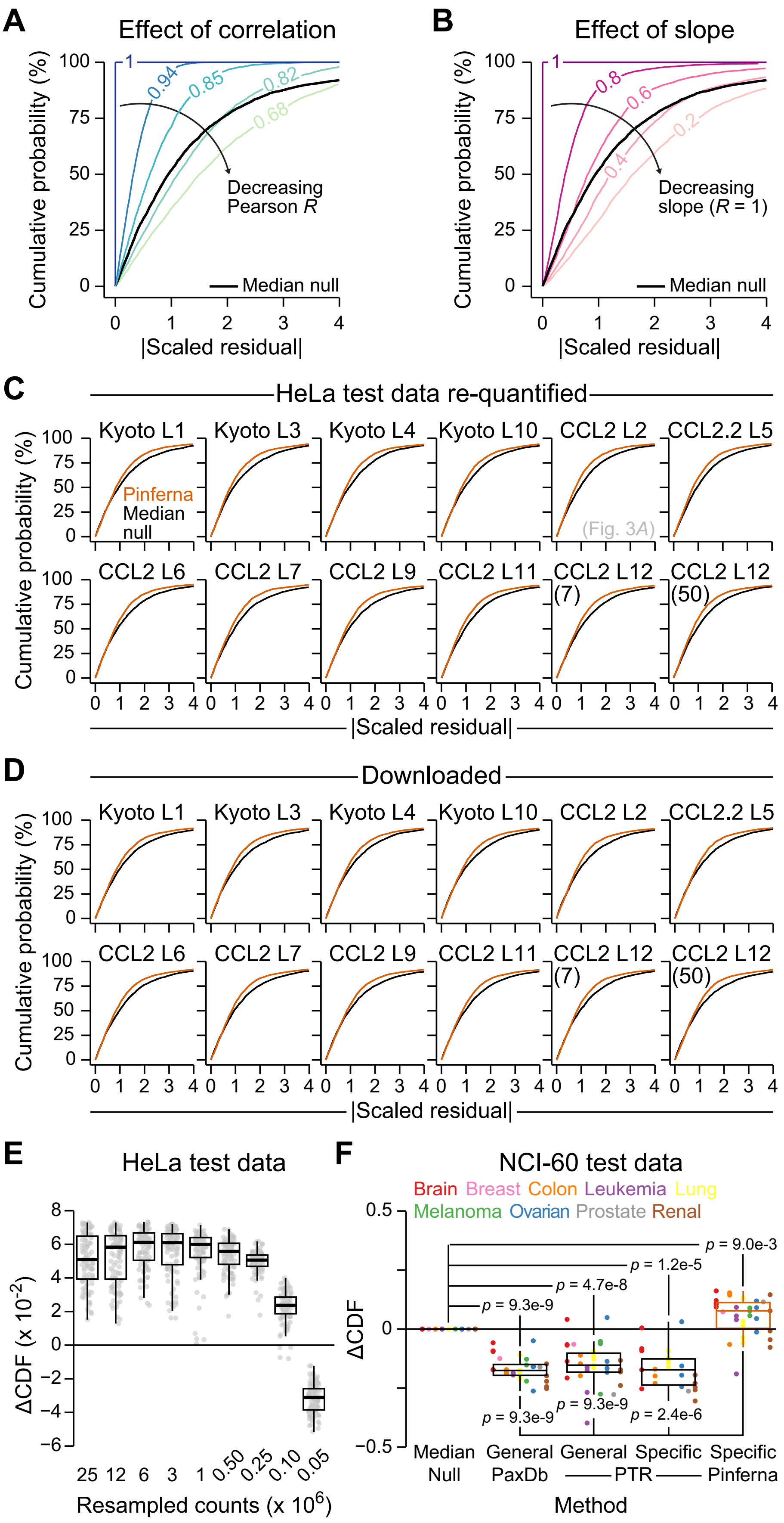

A
Effect of correlation
Cumulative probability (%)
|Scaled residual|
1
0.94
0.85
0.82
0.68
Decreasing Pearson R
Median null
B
Effect of slope
1
0.8
0.6
0.4
0.2
Decreasing slope (R = 1)
Median null
C
HeLa test data re-quantified
Kyoto L1
Kyoto L3
Kyoto L4
Kyoto L10
CCL2 L2
CCL2.2 L5
CCL2 L6
CCL2 L7
CCL2 L9
CCL2 L11
CCL2 L12 (7)
CCL2 L12 (50)
Pinferna
Median null
(Fig. 3A)
D
Downloaded
E
HeLa test data
ΔCDF (x 10-2)
Resampled counts (x 106)
F
NCI-60 test data
Brain Breast Colon Leukemia Lung
Melanoma Ovarian Prostate Renal
ΔCDF
p = 9.0e-3
p = 1.2e-5
p = 4.7e-8
p = 9.3e-9
p = 9.3e-9
p = 9.3e-9
p = 2.4e-6
Median Null
General PaxDb
General PTR
Specific PTR
Specific Pinferna
Method

Figure EV3. Robustness of Pinferna predictions.

(**A**, **B**) Cumulative distribution of scaled residuals degrades with decreasing correlation (**A**) and slope (**B**) between predicted and measured values. A synthetic dataset was created by taking the median TPM and protein value for each gene among 12 HeLa cell lines (Liu et al, 2019) and using perturbations of the synthetic data as a prediction. The median null for this dataset relative to Pinferna is shown for reference. To disrupt correlations (**A**), Gaussian white noise with increasing variance was added to the measured HeLa protein abundances to achieve the indicated Pearson correlations. Measured protein abundances were separately multiplied by the indicated slope while retaining a linear relationship (**B**). (**C**, **D**) Prediction accuracy is not dependent on SWATH analytical details. Cumulative distribution plots comparing Pinferna and median-null predictions for paired RNA-seq–SWATH datasets of 12 HeLa derivatives (PRJNA437150; PXD009273) named as in the publication (Liu et al, 2019). Proteins were re-quantified from raw SWATH data processed exactly like the meta-assembly ("Methods") (**C**); or, protein quantities were taken directly from the publication (Liu et al, 2019) (**D**). Results from the re-quantified CCL2 L2 derivative are reprinted from Fig. 3A. (**E**) Prediction accuracy is not heavily dependent on RNA-seq read depth. Count-based RNA-seq data for the HeLa lines was averaged and iteratively downsampled (gray) and TPM values re-estimated before making proteome-wide copy-number predictions with Pinferna. See Fig. 3B for an explanation of ΔCDF. (**F**) Prediction accuracy is not dependent on RNA-seq analytical details. Pinferna predictions were made using TPM values taken directly from the original publication (Reinhold et al, 2019), and ΔCDF values were calculated for NCI-60 cell lines excluded from model training (Fig. 1A) and organized by cancer type. Data information: For (**E**), $n = 100$ iterations. For (**F**), $n = 5$ brain, 1 breast, 3 colon, 4 leukemia, 4 lung, 3 melanoma, 3 ovarian, 1 prostate, 5 renal cell lines. Differences between groups were assessed by paired sign-rank tests with Šidák correction. For (**E**, **F**), box-and-whisker plots show the median ΔCDF (horizontal line), interquartile range (IQR; box), and an additional 1.5 IQR extension (whiskers) of the data.

© The Author(s)

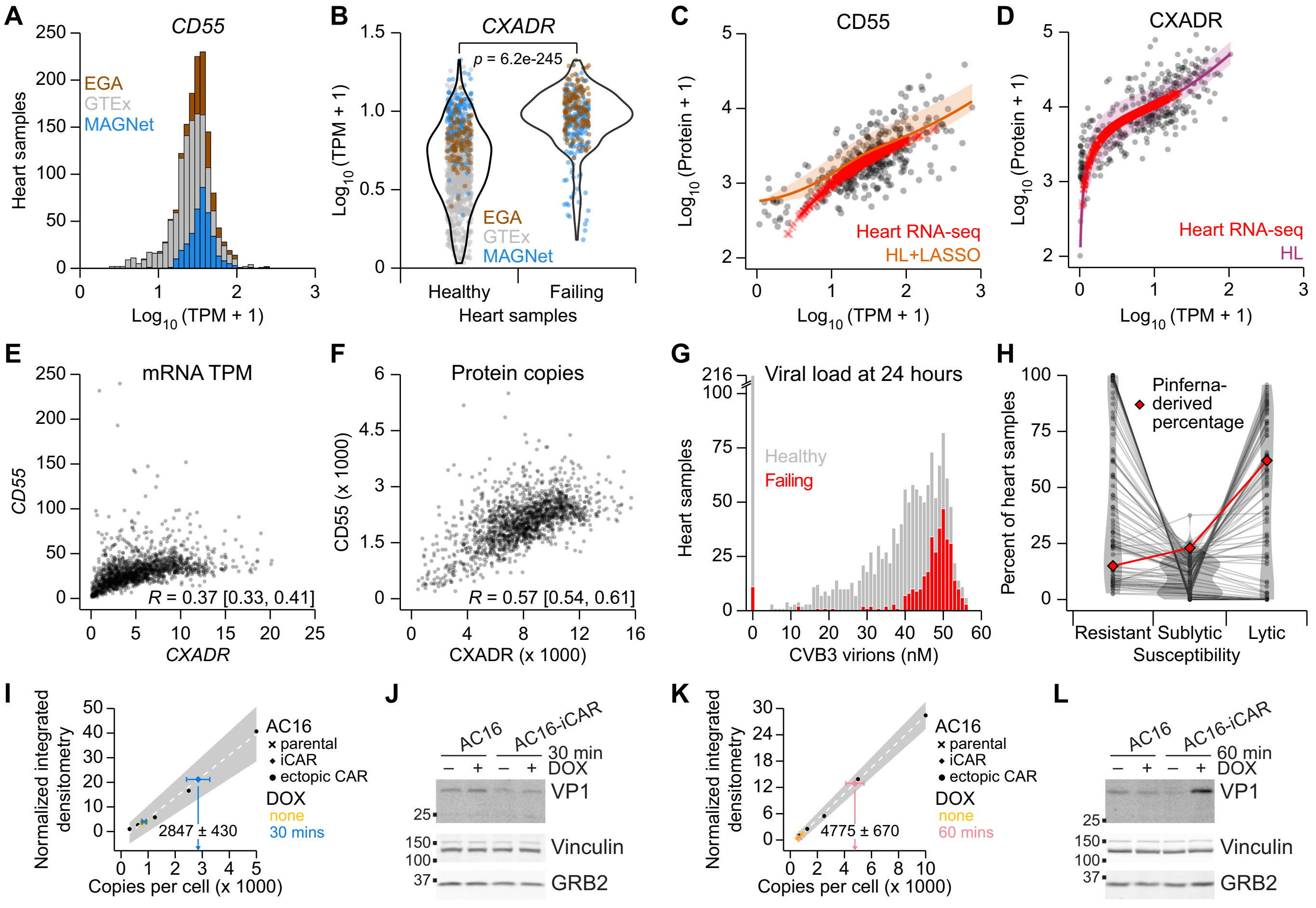

Figure EV4. Calibrated protein inferences of CD55 and CXADR yield disease-related predictions of coxsackievirus B3 (CVB3) susceptibility.

(**A**) Distribution of *CD55* abundance (Dataset EV7) separated by source data: EGA (brown; EGAS00001002454), GTEx (gray; phs000424.v9.p2), MAGNet (blue; GSE141910). (**B**) *CXADR* is upregulated in failing hearts. *CXADR* abundance (Dataset EV7) was stratified by heart health and colored by source data as in (**A**). (**C**, **D**) Calibration plots (orange (**C**); purple (**D**)) and Pinferna predictions for CD55 (**C**) and CXADR (**D**) in human heart samples (Heart RNA-seq; red). CD55 deviations from the smoothed best fit are caused by cardiac-specific features in the HL + LASSO regressions. (**E**, **F**) CD55–CXADR coregulation is increased at the protein level (**F**) compared to the mRNA level (**E**). (**G**) Replotted histogram of Fig. 4C separated by heart health (Dataset EV7). (**H**) Predicted prevalence of susceptibility groups based on randomized measurements. After linearly scaling to randomized abundances of CD55 and CXADR, CVB3 infections were simulated for 1489 heart samples as in Fig. 4B. The 24-h end states were quantified by the percentage of samples with resistant ([CVB3 virions] = 0 nM), sublytic (0 nM < [CVB3 virions] < 36 nM), and lytic ([CVB3 virions] ≥ 36 nM) phenotypes (Fig. 4C). Pinferna-derived percentages are overlaid in red. Results from the randomized simulations are connected, and densities in each group are shown by a violin plot in the background. (**I–L**) AC16 cardiomyocytes were stably transduced with doxycycline (DOX)-inducible CXADR-V5 (iCAR), induced for 30 min (**I**, **J**) or 60 min (**K**, **L**) and quantified for CXADR (**I**, **K**) or infected with CVB3 (multiplicity of infection = 5) for 6 h and immunoblotted for VP1 with vinculin and GRB2 as loading controls (**J**, **L**). Representative immunoblots of (**I**, **K**) are shown in Fig. 4H, J, and immunoblots of (**J**, **L**) are quantified in Fig. 4I, K. Data information: For (**B–F**), $n = 1489$ heart samples. For (**B**), differences between groups were assessed by rank-sum test. For (**C**, **D**), best-fit calibrations ± 95% confidence intervals are overlaid on the proteomic–transcriptomic data from $n = 369$ cancer cell lines. For (**E**, **F**), the Pearson R is shown with 95% confidence interval in brackets calculated by the Fisher Z transformation. For (**H**), $n = 100$ randomizations. For (**I**, **K**), $n = 4$ biological replicates calibrated against a five-point standard curve of AC16-CAR cells fit as a linear model.

© The Author(s)
